# Pathological Society of Philadelphia

**Published:** 1888-02

**Authors:** 


### PATHOLOGICAL SOCIETY OF PHILADELPHIA.

In an address before this society, by Dr. W. T. Councilman, of Baltimore, Maryland, on Some Further Investigations on the Malarial Germ of Lavaran, he said:

This organism, first described by Lavaran, has been met with in every case of malarial fever which the writer has met with. The organism is in high degree polymorphous, and ten tolerably distinct forms may be found in the blood. Some of these evidently represent different stages of development, and the connection between them is obvious. Others present such marked differences in form that no connection between them can be made out. Some of the forms are only found outside of the red corpuscles, and others are found free in the blood. They are, 1, non-pigmented small amœba-like bodies inside the red corpuscles; 2, pigmented bodies larger than No. 1, also in red corpuscles; 3, pigmented bodies about the size of red corpuscles; 4, segmenting forms of the No. 3 body; 5, small hyaline bodies, which are formed by this segmentation; 6, a crescent-shaped body with pigment in the centre, the horns of the crescent being often connected by a fine line; 7, round or oval bodies which differ from No. 6 in shape only; 8, a pigmented body provided with numerous long, actively moving flagellæ; 9, actively moving free flagellæ, which are evidently derived from No. 8; 10, a pigmented body with an active undulatory movement of its periphery. The first five forms are found only in intermittent fever, No. 4 only being seen in the blood during the chill period, and its presence is invariably connected with the chill; Nos. 6 and 7 are found in cases of malarial cachexia.

The most interesting forms, and about whose parasitic nature there can be no doubt, are the bodies Nos. 8 and 9. These are generally absent in blood taken from the finger, but they may be found in any type of the disease. They are the only forms of the organism whose presence in the blood is not associated with a special type of the disease. They were found, however, in fifteen out of the twenty cases in which the blood of the spleen was examined. Of these twenty cases twelve were cases of malarial cachexia, and eight of intermittent fever. In the twelve they were found ten times and in the eight cases of intermittent five times. From this it seems probable that Lavaran was right in considering the flagellate organism the most important form of the parasite. The influence of quinine on the intracorpuscular forms of the parasite is most marked. Doses of 15 grs. t. i. d., for two days in succession, were found sufficient to cause them to disappear. The effects of the quinine were not so apparent in the other forms. The crescents were apparently not diminished in number in one individual after he had taken 45 grs. of quinine daily for seven days, and 60 grs. daily for four days.

Professor Wm. Osler said the thought which had struck him most forcibly, in looking over this subject, was the almost perfect unanimity which has prevailed among the different observers as to the appearance of these organisms. With the sole exception of the segmented form (No. 4),

Lavaran and the early observers had described them all. His own observations, since the communication he had presented to the Society last year, had been somewhat limited. He had, however, made a series of observations upon the blood of fishes and birds, since it had been stated that bodies resembling Nos. 1, 2, and 3 had been found in the blood of carp and some water-fowl. Professor Baird had offered him facilities for this work at Wood's Holl, and had kindly furnished him with forty-five carp. He had failed to detect any such organisms in the blood of these. In the blood of a goose sent him from Ontario he had found one or two pigmented bodies. It had been stated by Dr. McCallum, who sent him the goose, that the bird had malaria. However, the bodies were not numerous, nor was the temperature of the goose elevated, nor, so far as he could make out, had it chills. Dr. Councilman had not figured one body that is very peculiar indeed, namely, a solid body in the centre of a clear space. It stains like a micro-organism, varies in size, and although the body itself does not change in form, yet there are sometimes changes in outline in the clear space surrounding it. They were somewhat abundant in one case only.

With regard to the clear bodies (No. 1), he said, in five or six instances he had seen such bodies pass out from the corpuscle, remaining out and undergoing no further change of form. He was not altogether prepared to say what was the relationship of these bodies to the other bodies described. It has been claimed that similar changes can be obtained by special methods of treating the blood. The most important question is first to determine the relationship of the hyaline to the pigmented bodies, and the possibility that the hyaline may not be directly associated with them. He was convinced that the pigmented and segmented bodies were merely different stages. He could fully confirm what Dr. Councilman said with regard to the crescents. They are most peculiar and interesting bodies, occurring in the chronic cases and in those in which there had been no chills. Three weeks ago he had diagnosticated a case as one of mild typhoid fever; it had lasted eight or ten days, with constant fever, up in the evening, down in the morning; slight enlargement of the spleen, no spots. His resident examined the blood and found what he thought were crescents. The case got rapidly better, left the hospital, and returned in a few days with a distinct chill, with crescents in the blood and a well-marked remittent fever. The mobile forms he had not seen nearly as frequently as Dr. Councilman; though he had not examined the blood from the spleen they had been present in eight or ten cases. Nor had he seen free filaments nearly so often; when he wrote his paper he had not seen them at all. Since then he had watched the process of separation. It was out of the question to suppose that the crescents or mobile forms could come from degeneration in the stroma of the corpuscle, but that the hyaline forms resulted from such changes was not altogether improbable; and further investigations were necessary to determine this point.

Dr. J. P. C. GRIFFITHS called attention to the diagnostic value of these organisms, and instanced a case where, from the indefinite history and symptoms, he was unable to make a diagnosis until after an examination of the blood, when a short course of treatment resulted in recovery.

Professor H. C. WOOD said that no one seemed to have made any connection between the crescents and the amœboid forms; they seem to differ in that these are destroyed by quinine, those are not affected. We know that malarial cachexia is cured by quinine, arsenic, and iron. If these remedies have no effect on the crescents, what connection have these bodies with malaria, and what becomes of them--do they eventually disappear?

Dr. FORMAD asked if these organisms are the same as described by Hütter some twenty years ago.

Dr. COUNCILMAN said, in conclusion, that Hütter described moving bodies attacking the red corpuscles, existing in all fevers and apparently almost everywhere else. These observations had never been confirmed. The point raised by Dr. Wood had always puzzled him, and for a long time he had tried to reconcile himself to a belief in two distinct diseases, but this he could not do, as always as the other forms disappear the crescents appear. He had never seen the crescents unless with a history of previous chills. He was not altogether prepared to say that quinine had no effect on the crescents, though in several cases he had given it in large doses with no results. Still in some cases they do seem to disappear. He thought with Dr. Osler that the crescents could not be possibly produced by changes in the stroma of the corpuscles, though some of the other forms might.

